# M. Gunn, M. D.

**Published:** 1849-08

**Authors:** 


*M. Gunn, M. D.*, has been appointed by the Regents of the University of Michigan, Professor of Anatomy in the Medical department, located at Ann Arbor.

Dr. Gunn is a young man, but he has already distinguished himself by his zeal in his profession, and especially in the department to which he has now been appointed. His medical studies were commenced under the pupilage of one of the best anatomists and most persevering students of Western New York, Dr. Carr, of Canandaigua; whose office had already furnished two Professors of Anatomy, in respectable Medical Colleges, both of whom have become deservedly eminent.

He graduated at Geneva Medical College, and during a part of his course officiated as adjunct Demonstrator. From his enthusiasm in the dissecting room, and his fluency and readiness as a demonstrator whenever the absence of Dr. Ford rendered his performance of these duties necessary, it was confidently predicted that he would early command the attention of the colleges, and that he would fill any chair of anatomy with honor. His *thesis* on the anatomy and physiology of the sympathetic nerves, was characterized by great originality and extensive research.

We congratulate the University of Michigan upon this fortunate accession to the talent, and respectability of its medical corps, and while the Trustees continue to give evidence of so much judgment and impartiality in the selection of their officers, we wish them, what they will certainly deserve, eminent success. F. H. H.

